# Novel plant flavonoid electrochemical sensor based on in-situ and controllable double-layered membranes modified electrode

**DOI:** 10.1371/journal.pone.0237583

**Published:** 2020-08-17

**Authors:** Jing Hu, Renjie Zhou, Hongwei Lin, Qiuyuan Wei, Feilong Hu, Xin Yang

**Affiliations:** 1 Huaihua Key Laboratory for Preparation of Ceramics Materials and Devices, Hunan Engineering Laboratory for Preparation Technology of Polyvinyl Alcohol Fiber Material, Huaihua University, Huaihua, PR China; 2 Guangxi Key Laboratory of Chemistry and Engineering of Forest Products, Guangxi University for Nationalities, Nanning, PR China; 3 Key Laboratory of Research and Utilization of Ethnomedicinal Plant Resources of Hunan Province, Key Laboratory of Hunan Higher Education for Western Hunan Medicinal Plant and Ethnobotany, Huaihua University, Huaihua, PR China; Qatar University, QATAR

## Abstract

Identification and quantification of plant flavonoids are critical to pharmacokinetic study and pharmaceutical quality control due to their distinct pharmacological functions. Here we report on a novel plant flavonoid electrochemical sensor for sensitive and selective detection of dihydromyricetin (DMY) based on double- layered membranes consisting of gold nanoparticles (Au) anchored on reduced graphene oxide (rGO) and molecularly imprinted polymers (MIPs) modified glassy carbon electrode (GCE). Both rGO-Au and MIPs membranes were directly formed on GCE via in-situ electrochemical reduction and polymerization processes step by step. The compositions, morphologies, and electrochemical properties of membranes were investigated with X-ray powder diffractometry (XRD), Fourier transform infrared spectrum (FTIR), Field emission scanning electron microscopy (FESEM) combined with various electrochemical methods. The fabricated electrochemical sensor labeled as GCE│rGO-Au/MIPs exhibited excellent performance in determining of DMY under optimal experimental conditions. A wide linear detection range (LDR) ranges from 2.0×10^−8^ to 1.0×10^−4^ M together with a low limit of detection (LOD) of 1.2×10^−8^ M (*S/N* = 3) were achieved. Moreover, the electrochemical sensor was employed to determine DMY in real samples with satisfactory results.

## Introduction

Dihydromyricetin (DMY) is the most bioactive and abundant plant flavonoid found in the leaves of Ampelopsis grossedentata (a traditional herbal tea in Huaihua region of China and its alternative name is vine tea). The health beneficial properties of DMY cover anticancer, antimicrobial, antioxidant, anti-inflammatory, antidiabetic, and neuroprotective effects. Therefore, the identification and quantification of it, and investigation of the biological activities have aroused increasing awareness [1, 2]. At present, identification and quantification of DMY mainly focus on chromatographic and spectral techniques including high-performance liquid chromatography (HPLC) [3], high-speed countercurrent chromatograph (H-SCC) [4], LC-mass spectrometry (MS)/MS (LC-MS/MS) [5], and fluorescence spectroscopy (FS) [6]. Unfortunately, expensive instruments or complicated pretreatments are usually required in these time-consuming and skills-training techniques.

Compared with chromatographic and spectral techniques mentioned above, electrochemical techniques (ETs) possess advantages of simplicity, celerity, sensitivity, and inexpensive. Furthermore, the ETs can help to identify the redox of drug compound and offer significant information related to pharmacokinetic. Thus, interests in analysis of flavonoids by applying ETs have been increasing. However, few reports are related to voltammetric and electrochemistry determination of DMY up to now. For instance, Xu and Zou fabricated the DMY voltammetric sensors based on glassy carbon electrode (GCE) modified with SWCNs dispersed in nafion and DNA immobilizing on En/PGA [7, 8]. The sensitive and simple electrochemical sensor for determining DMY based on graphene (Gr)-nafion film modified GCE was constructed by Wang and his group [9]. Beyond these, there are no other reports.

Currently, more and more researches are being made efforts to provide novel modifiers toward the electrochemical sensors with better sensitivity and selectivity. Extensive studies indicated that Gr and its derivative reduced graphene oxide (rGO) are promising and advanced carbon based nano-materials. Moreover, the stupendous features of large surface area, high stability, and excellent mobility of charge carriers make them ideal platforms for anchoring various noble metal particles in the fields of electrochemical sensing. In particular, rGO supported gold nanoparticles (Au) provide amplified electrical conductivity performance owe to the acceleration of charge transfer from substrates to nanoparticles. The hybridization between the sp^2^ dangling bonds of rGO resident defect sites and Au is beneficial to improve stability of nanocomposite [10–12]. Many researches make the combination of them as electrode modifiers for detection of hydrogen peroxide, dopamine, NADH, methylparaben, and aflatoxin B1 [10–14], etc. Due to good recognition and enrichment abilities to target molecules together with many other advantages including stability, inexpensive, simplicity, and reusability compare with natural biological receptors, molecularly imprinted polymers (MIPs) have been regarded as ideal artificial recognition elements to improve the measuring selectivity for electrochemical sensor in complicated matrices [15–17]. Numerous MIPs modified electrodes were fabricated and used widely in determination of quercetin, amyloid-β protein, chlorpyrifos, and azithromycin [18–21], etc. However, drawbacks of slow mass transfer, low binding capacity, and poor binding kinetics resulted from these organic materials. The challenges are expected to be solved by incorporating high conductivity nanomaterials. To the best of our knowledge, there are no reports based on neither rGO-Au nanocomposite nor MIPs modified electrode to quantitative analysis of DMY till now.

Based on our previous researches in Gr-based nanocomposite and MIPs [10, 11, 18], this investigation aims at fabricating a novel DMY electrochemical sensor based on double-layered membranes consisting of rGO-Au nanocomposite and MIPs modified GCE (labeled as GCE│rGO-Au/MIPs). Both rGO-Au and MIPs membranes were formed on GCE via in-situ electrochemical reduction and polymerization processes step by step. Compared with previously published electrochemistry methods for DMY detection, the strategies involved in electrode modifying procedures have following advantages: (i) the electrochemical reduction and polymerization processes are not only in-situ but also controllable, facile, and rapid, avoiding complex and time-consuming modification processes. (ii) the usage of precursors such as graphene oxide (GO), gold (III) chloride trihydrate (HAuCl_4_**·**3H_2_O), and acrylamide (AM) is nearly 100%, reducing the waste of reagents. (iii) no hazardous reducing chemicals or extensive organic solvents was used, and the approach is environmental friendly. Attributing to reasonable synergistic effects of double-layered membranes, the GCE│rGO-Au/MIPs electrode showed superior sensitivity and selectivity. Meanwhile, the electrochemical sensor was employed to determine DMY in real samples with high accuracy and recovery, which holds great potential application in the future.

## Experimental section

### Instruments and reagents

The crystallization degrees and phase purity of composites were characterized with Rigaku Ultima IV X-ray powder diffractometer (XRD). The samples with KBr pellets were determined by Shimadzu IR prestige-21 Fourier transform infrared spectroscopy (FTIR). The morphologies characterization of different electrodes were carried on Zeiss Sigma HD Field emission scanning electron microscopy (FESEM) connected to Oxford instruments X-Max^N^ energy-dispersive X-ray spectrometer (EDS). Under room temperature and N_2_ protection, all electrochemical experiments were performed on CH Instruments CHI 660E electrochemical workstation (EW). The EW employed with modified/bare GCE (Φ:3 mm), saturated calomel electrode (SCE), and platinum wire electrode as working, reference, and counter electrodes, respectively. The determination of DMY in Ampelopsis grossedentata samples was also carried out on Shimadzu LC-20AT HPLC.

DMY (≥98%, HPLC) was supplied by Sigma-Aldrich (USA). The stock solution of DMY was stored at 4 °C darkly and diluted just before use. The Ampelopsis grossedentata was purchased from Laifeng Jinqi Tengcha Bio Co., Ltd (Enshi, China). The plant materials were identified based on plant morphology by Dr. Zhaotun Hu in the College of Biological and Food Engineering, Huaihua University (Huaihua, China). Graphite powder was obtained from XFNANO Materials Tech Co., Ltd (Nanjing, China). Nafion solution used in membranes formation (0.5 wt.%), HAuCl_4_·3H_2_O with purity of 99.99%, AM, azobisisobutyronitrile (AIBN), ethylene glycol dimethacrylate (EGDMA), and other chemicals were provided by Sinopharm Medicine Holding Co., Ltd (Shanghai, China). Various proportions of NaH_2_PO_4_ (0.1 M) and Na_2_HPO_4_ (0.1 M) were mixed to obtain different pH values phosphate buffer (PB) electrolyte solutions.

### Fabrication of modified electrodes

The GCE│rGO-Au electrode was fabricated via an in-situ electrochemical reduction with precursors of GO and HAuCl_4_**·**3H_2_O simultaneously [22]. Firstly, the traditional Hummers method was employed to prepare GO with graphite powder [23, 24]. 0.5 mg of ground GO and 1.0×10^−5^ mol of HAuCl_4_**·**3H_2_O were dissolved in 1.0 mL of 0.5 wt.% nafion solution to prepare the homogenous dispersion. Then, 6.0 μL of dispersion was cast onto polished GCE and heated by an infrared lamp till dry. Finally, The formation of rGO-Au nanocomposite on GCE was achieved by employing a potential at -1.0 V (*vs*. SCE) in H_2_SO_4_ (0.1 M) for 600 s. In control experiments, rGO and Au were formed on GCE by applying same electrochemical reduction approach under similar experimental conditions (labeled as GCE│rGO and GCE│Au).

The DMY-MIPs polymer membrane modified GCE│rGO-Au was fabricated by an in-situ polymerization method [25]. 1.0 mL of porogen solvent (acetonitrile) was added with 0.1 mmol of functional monomer (AM) and 0.025 mmol of template molecule (DMY), and dissolved with ultrasonic. After that, 0.08 mmol of initiator (AIBN) and 1.5 mmol of cross-linking agent (EGDMA) were added. The polymerization solution was deoxygenated by bubbling N_2_ for 15 min. 5.0 μL of the mixture was uniformly coated onto GCE│rGO-Au electrode and heated by an infrared lamp till a dry and transparent membrane appeared. Finally, the modified electrode was immersed into an acetic acid/methanol mixture solution (*v/v =* 1:1) for 30 min to extract the template molecules of DMY from imprinted membrane and obtained the GCE│rGO-Au/MIPs electrode. Under same conditions in absence of DMY, non-imprinted membrane modified GCE│rGO-Au was prepared (labeled as GCE│rGO-Au/NIPs). All modified electrodes were stored in dry and ambient condition for further electrochemical measurements.

### Electrochemical measurements

**Fig 1:**
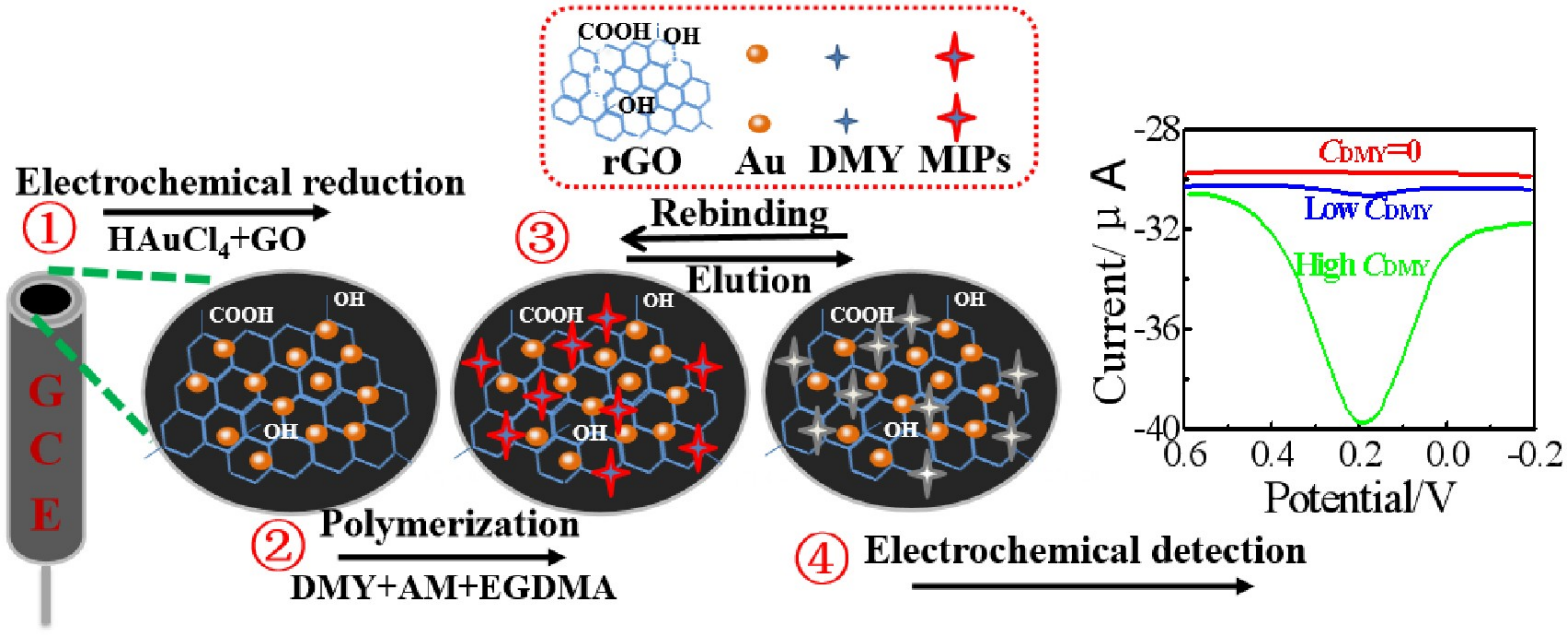
Schematic diagram for fabrication procedures of GCE│rGO-Au/MIPs.

The electrochemical performances of different electrodes were investigated by electrochemical impedance spectroscopy (EIS, Applied potential: +0.2 V. Frequency range: 0.01–100 K Hz. Amplitude: 5 mV), cyclic voltammetry (CV, Low E: -1.0 V. High E: +1.0 V. Scan rate: 100 mV**·**s^-1^. Sample interval: 0.001 V), and differential pulse voltammetry (DPV, Init E: -0.2 V. Final E: +0.6 V. Amplitude: 0.05 V). After each run, the GCE│rGO-Au/MIPs electrodes were eluted with acetic acid/methanol mixture solution for 30 min to make sure no redox currents of the modified electrode were observed, confirming the total removal of DMY from imprinted membrane and obtaining the reusable electrodes. The schematic diagram for fabrication of modified electrode is shown in Fig 1. The real samples of Ampelopsis grossedentata after pretreatments were followed the described procedures as recommended above.

## Results and discussions

### Characterization of membranes

**Fig 2:**
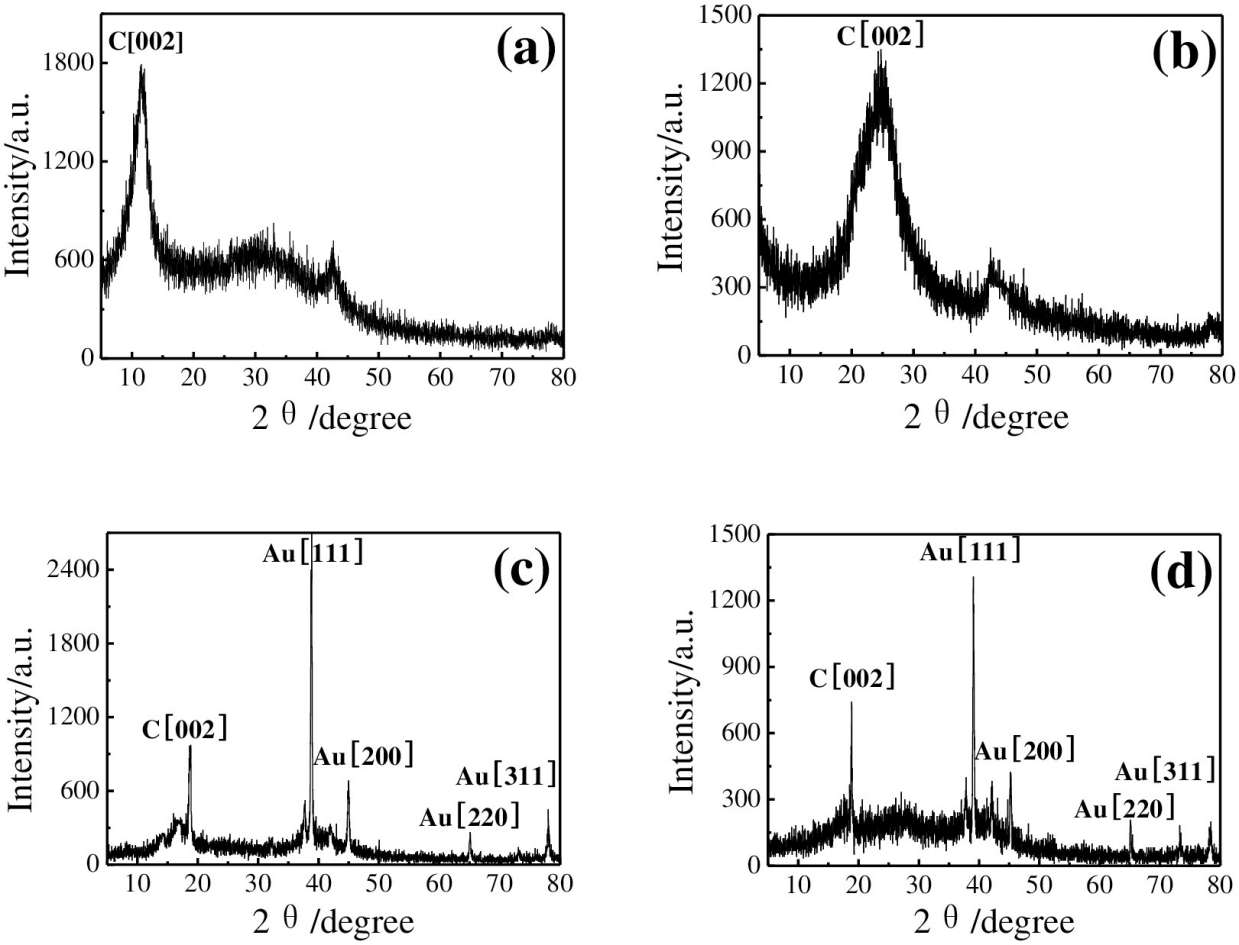
XRD spectra of GO (a), rGO (b), rGO-Au (c), and rGO-Au/MIPs (d).

The XRD spectra of GO, rGO, rGO-Au, and rGO-Au/MIPs are showed in Fig 2. After oxidation, characteristic peak at 2θ = 26° corresponding to graphite disappeared and was substituted for a distinct peak of GO at 2θ = 10.8° (Fig 2a) [26]. The (002) peak at 2θ = 26° exhibits the crystalline feature of Gr, indicating the formation of rGO via an electrochemical reduction of GO (Fig 2b) [27]. The diffraction peaks related to rGO (002) and Au (311), (220), (200), (111) can be observed obviously in rGO-Au nanocomposite (Fig 2c) [22]. The results imply the in-situ electrochemical reduction strategy is efficient for simultaneous formation of rGO-Au nanocomposite. No peaks associate with impurities were found in rGO-Au/MIPs composites (Fig 2d).

**Fig 3:**
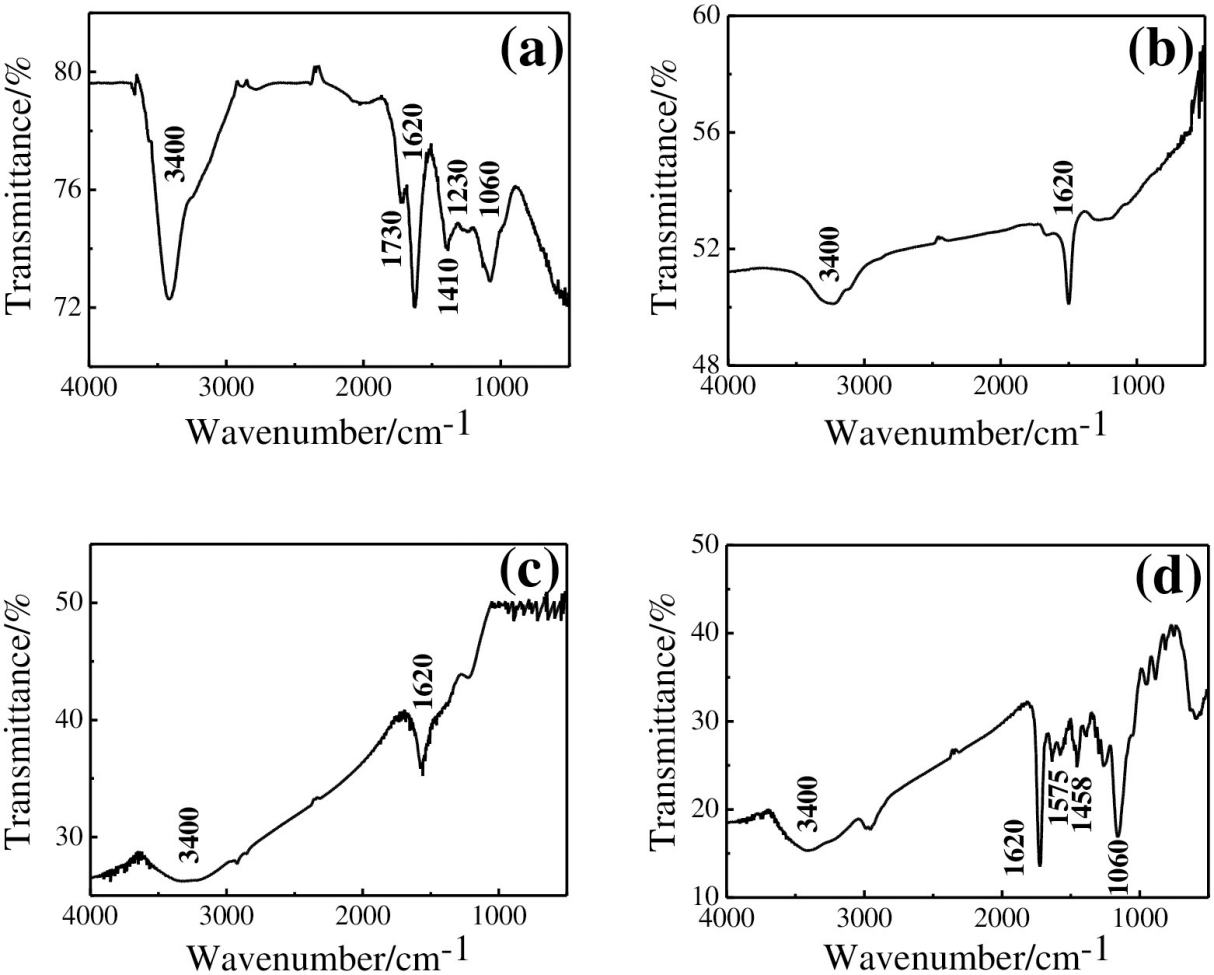
FTIR spectra of GO (a), rGO (b), rGO-Au (c), and rGO-Au/MIPs (d).

The FTIR spectrum of GO displays alkoxy (1060 cm^-1^), aromatic C = C (1620 cm^-1^), carboxyl C-O (1410 cm^-1^), C = O (1730 cm^-1^), epoxy C-O (1230 cm^-1^), and -OH (3400 cm^-1^) (Fig 3a) [28]. The remained skeletal vibration at 1620 cm^-1^ related to Gr and disappearance of most oxygen-containing groups in Fig 3b indicate formation of rGO [24]. The absorbance bands of rGO-Au were little changed (Fig 3c). Many adsorption peaks located between 2000 cm^-1^ and 1000 cm^-1^ presence in spectrum of rGO-Au/MIPs (Fig 3d) can be classified to the organic groups introduced by DMY, EDGMA, and AM in MIPs [18].

**Fig 4:**
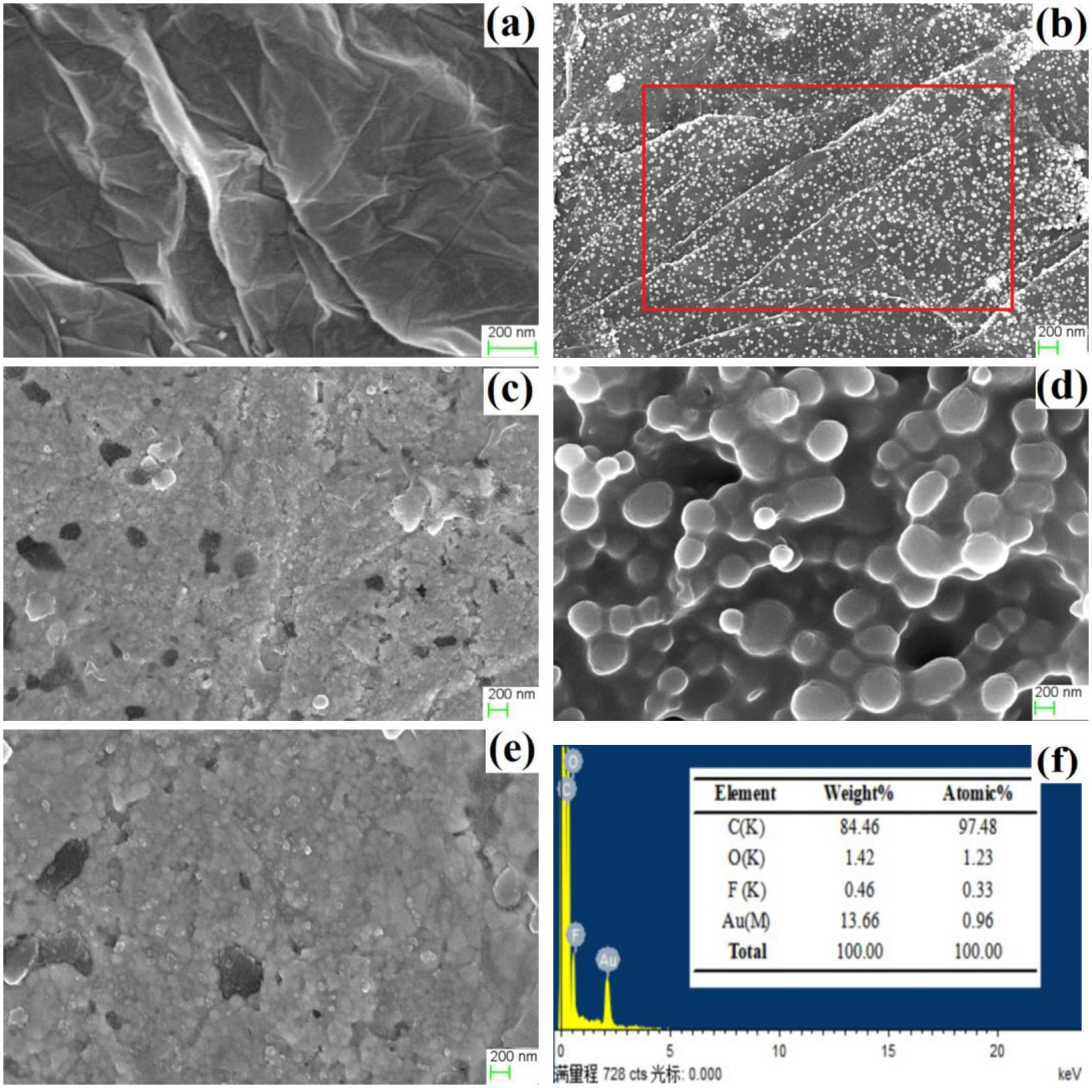
FESEM of GCE│rGO (a), GCE│rGO-Au (b), GCE│rGO-Au/NIPs (c), GCE│rGO-Au/MIPs before (d), after (e) template removal, and EDS of marked area in FESEM image of GCE│rGO-Au (f).

The continuous and crumpled nanosheets of rGO appeared on GCE (Fig 4a), which are beneficial for maintaining large surface area and anchoring Au. Massive uncapped Au nanoparticles with particle size about 60 nm were uniformly anchored on rGO nanosheets (Fig 4b). The detectable elements shown in EDS spectrum of rGO-Au nanocomposite including C, O, F, and Au (Fig 4f). Most of C originate from rGO. The F and O mainly come from the nafion and functional groups of oxygen- containing in rGO, respectively. The peak at 2.1 keV belongs to Au. Both rGO and Au can amplify electrical conductivity and enlarge specific surface area. The morphologies among GCE│rGO-Au/NIPs (Fig 4c), GCE│rGO-Au/MIPs before (Fig 4d), and after (Fig 4e) template removal are different. The MIPs membrane became rougher after templates removal compared with before templates removal and non-imprinted membrane. The rougher imprinted membrane is good for enhancing rebinding efficiency and improving response selective [25].

### Characterization of electrochemical performances

**Fig 5:**
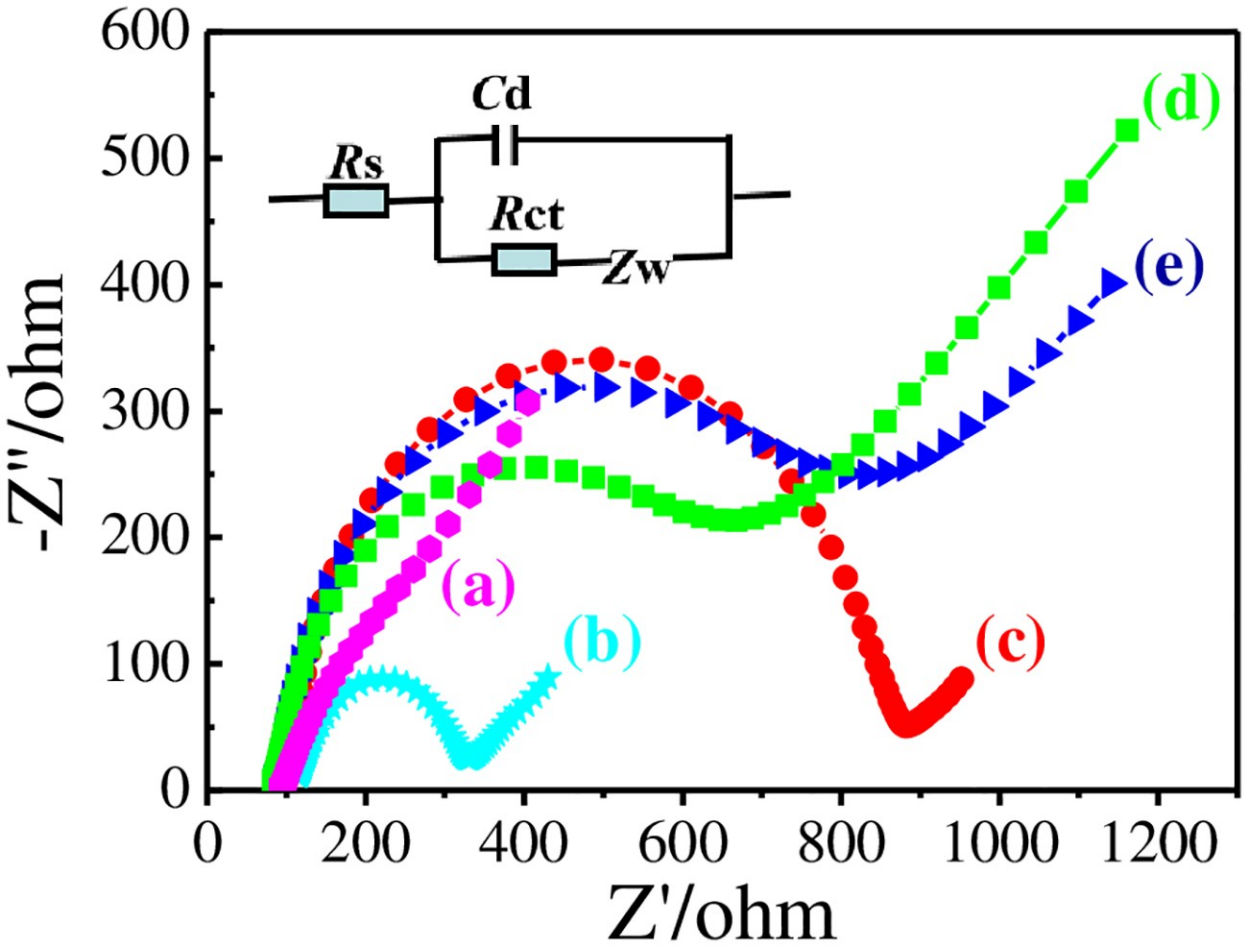
EISs of 0.1 M KCl solution containing 10 mM K_3_[Fe(CN)_6_]^3-/4-^ on GCE (a), GCE│rGO-Au (b), GCE│rGO-Au/MIPs before (c), after elution (d), and GCE│rGO-Au/NIPs (e).

The interfacial properties of 0.1 M KCl solution containing 10 mM K_3_[Fe(CN)_6_]^3-/4-^ as probes on different electrodes were studied with EIS (Fig 5). Randles equivalent circuit model in inset was used to fit EIS results, in which *R*ct indicates the interfacial electron-transfer impedance of probes on electrode surface and *R*ct changes with various membranes modified onto GCE. The GCE showed the smallest semicircle diameter and a negligible *R*ct (10 Ω) (Fig 5a). Due to the nafion in membrane blocks the [Fe(CN)_6_]^3-/4-^ diffusion though rGO-Au nanocomposite is electrical conductive, the *R*ct of GCE│rGO-Au electrode (Fig 5b) increased a little (330 Ω) [24]. The *R*ct (881 Ω) increased remarkably after modified with MIPs before elution of templates (Fig 5c), showing that the MIPs has been successfully immobilized on the membrane of rGO-Au. The non-conductive MIPs membrane forms barrier between solution and electrode, and blocks exchange of electrons [20]. The *R*ct (649 Ω) decreased a little after elution of templates (Fig 5d), showing that the templates were removed and the cavities formed. The *R*ct (812 Ω) located between MIPs before and after elution of DMY when immobilized rGO-Au/NIPs with similar process (Fig 5e). The phenomenon can be interpreted that the NIPs hinder electron exchange while the absence of DMY can accelerate electron exchange [18]. The results demonstrated that double-layered membranes of rGO-Au and MIPs have been step by step immobilized on GCE.

**Fig 6:**
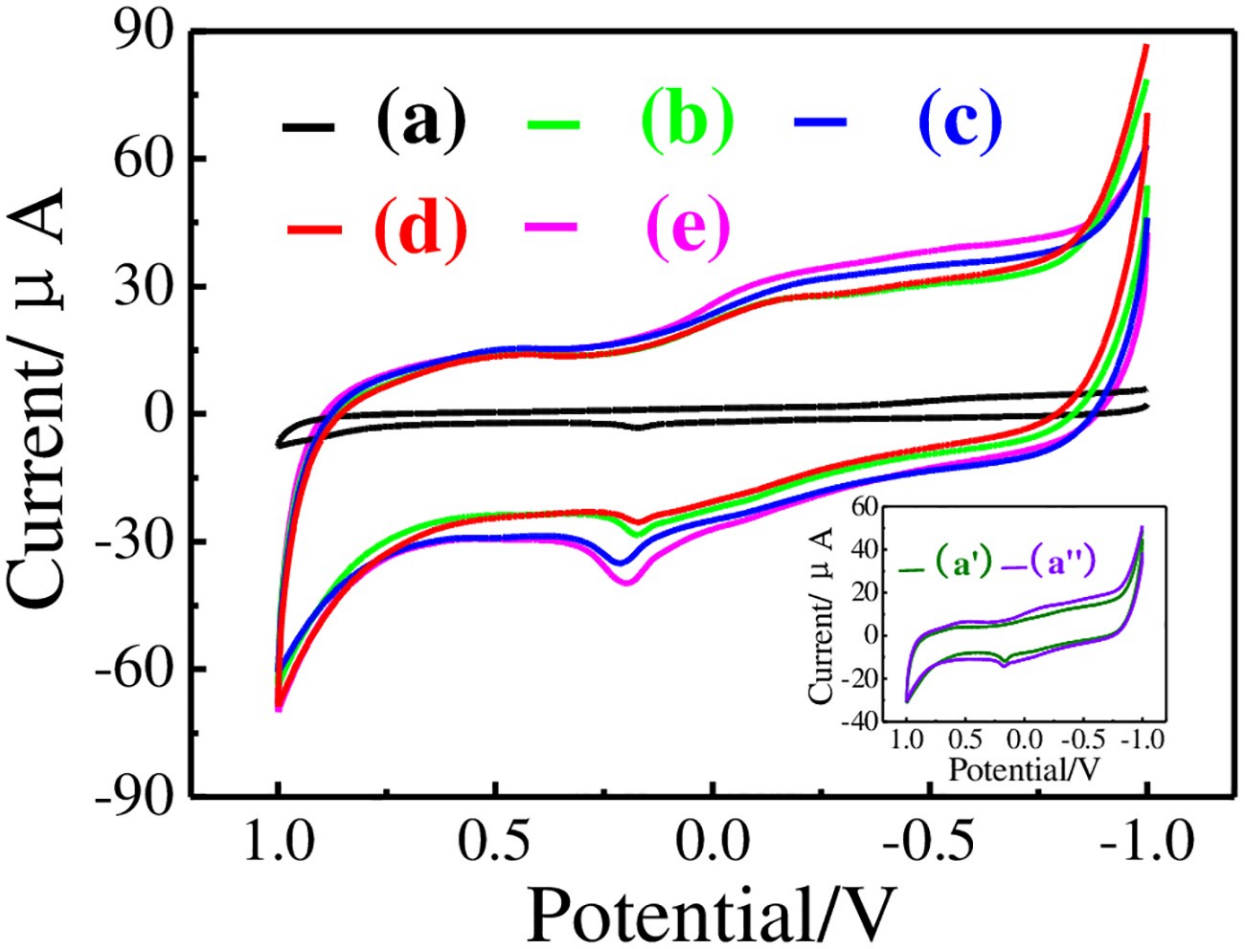
CVs of 20.0 μM DMY on GCE (a), GCE│rGO (a’), GCE│Au (a”), GCE│rGO-Au (b), GCE│rGO-Au/MIPs (c), GCE│rGO-Au/NIPs (d), and (c) added with 40.0 μM DMY (e).

The electrochemical behaviors of 20.0 μM DMY in pH = 6.0 PB recorded by different electrodes are shown in Fig 6. Generally, the electrochemical reaction of DMY can be appeared as an irreversible anodic peak current (Ipa) on all electrodes. The lowest Ipa for DMY on GCE was found at+0.19 V (Fig 6a). Both Ipa and background current increased on GCE│rGO, GCE│Au and GCE│rGO-Au electrodes (Fig 6a’, 6a” and 6b). These results can be deduced to the immobilized of rGO, Au, and rGO-Au nanocomposite on GCE are highly conductive, which affords porous structure for electron exchanging of DMY [10]. The Ipa of GCE│rGO-Au/MIPs electrode (Fig 6c) is larger than GCE, GCE│rGO, GCE│Au, and GCE│rGO-Au electrodes, indicating that MIPs has adsorption capacity toward DMY. However, the response of GCE│rGO-Au/NIPs electrode (Fig 6d) is lower than GCE│rGO-Au electrode but still higher than GCE. This can be explained by the synergistic effect of rGO-Au nanocomposite in current amplification exceeds the electron hinder effect of NIPs in current reduction. In addition, the Ipa of GCE│rGO-Au/MIPs electrode increased with higher DMY concentration (Fig 6e). These results confirm the potential application of GCE│rGO-Au/MIPs electrode in DMY analysis.

### Optimization of the experimental parameters

#### Volume of GO-HAuCl_4_·3H_2_O-nafion dispersion

The volume of GO-HAuCl_4_**·**3H_2_O-nafion dispersion on GCE is an important parameter for determining DMY. Several GCE│rGO-Au electrodes were prepared by casting 2.0 μL, 4.0 μL, 6.0 μL, 8.0 μL, and 10.0 μL of dispersion onto GCE, and electrochemical reduction in 0.1 M H_2_SO_4_ for 600 s. The Ipas of 20.0 μM DMY increased with the volume of dispersion modified onto GCE reached to 6.0 μL. More dispersion resulted in low peak currents. Hence, 6.0 μL of GO-HAuCl_4_**·**3H_2_O-nafion dispersion is fit for fabricating the GCE│rGO-Au electrode.

#### Time of electrochemical reduction

**Fig 7:**
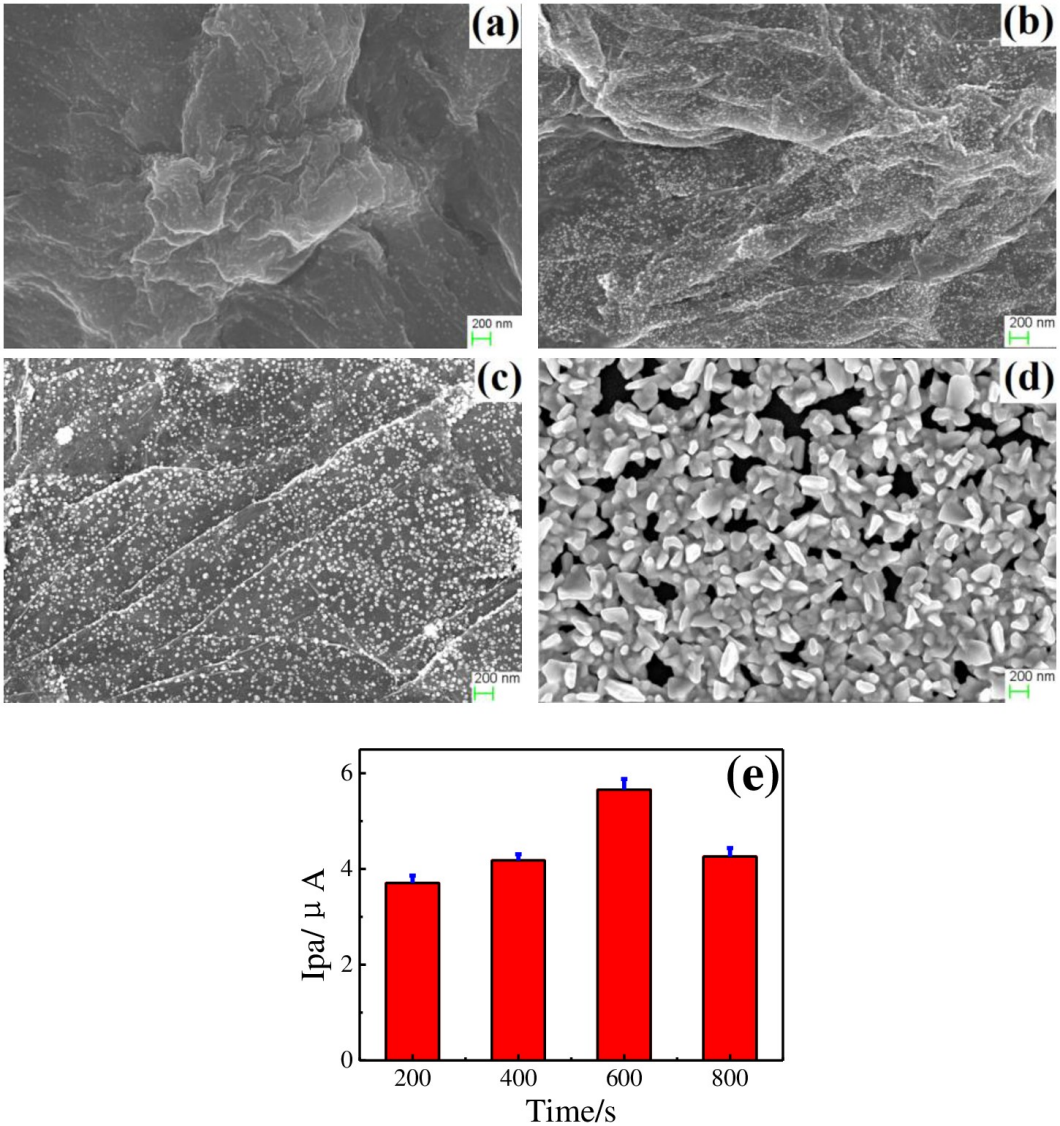
FESEM of GCE│rGO-Au with electrochemical reduction time of 200 s (a), 400 s (b), 600 s (c), 800 s (d), and their electrochemical performances toward 20.0 μM DMY (e).

Different sizes of Au anchoring on rGO sheets with different electrochemical reduction time and their electrochemical performances toward 20.0 μM DMY are shown in Fig 7. With reduction time of 200 s, the smallest size of Au about 20 nm and a low coverage on GCE was observed (Fig 7a). The size of Au successively grew to 40 nm (Fig 7b), 60 nm (Fig 7c), and 100 nm (Fig 7d) as time raised up to 400 s, 600 s, and 800 s. The GCE│rGO-Au electrodes with different electrochemical reduction time displayed distinct performances toward 20.0 μM DMY (Fig 7e). The increases of reduction time from 200 s to 600 s resulted the increases of Ipas and the longer time of 800 s induced a worse sensing performance. As is known to all, higher coverage of Au and rGO modified onto electrode, larger available surface area and more active sites for DMY adsorbing and reacting can be provided. The Au tended to form a compact surface as time increased up to 800 s, causing the lower current. Therefore, the reduction time of 600 s was selected.

#### Volume of polymerization mixture

The determination selectivity can be improved by the MIPs membrane as the target molecules of DMY can absorbed and enriched onto modified electrodes. However, the organic materials of MIPs are non-conductive. Five GCE│rGO-Au/MIPs electrodes were prepared via casting 1.0 μL, 3.0 μL, 5.0 μL, 7.0 μL, and 9.0 μL of polymerization mixture. The Ipas of 20.0 μM DMY were enhanced with the volumes of polymerization mixture cast onto GCE│rGO-Au electrode reached to 5.0 μL. More polymerization mixture resulted in decreased peak currents. Therefore, 5.0 μL of polymerization mixture is suitable to fabricate the GCE│rGO-Au/MIPs electrode.

#### Time of incubation and elution

The corresponding response currents of GCE│rGO-Au/MIPs electrode were recorded by DPV responses when it was immersed in PB containing 20.0 μM DMY with different time. The results indicated the Ipa reached the maximum value as the incubation time extended to 5 min, suggesting the fabricated sensor possesses a quick rebinding dynamics and the adsorption equilibrium can be achieved in short time.

Complete removal of templates from imprinted membrane after each run is important to obtain satisfactory selectivity results. Since H-bond is the major binding force between the templates and imprinted membrane, the acetic acid/ methanol mixture solution (1:1, v/v) was selected as eluent for removing DMY [25]. The results showed the Ipas of 20.0 μM DMY decreased gradually with increasing elution time till 30 min. The Ipa currents changed little with further increased elution time. Hence, 30 min was chosen as elution time.

#### pH value of PB

**Fig 8:**
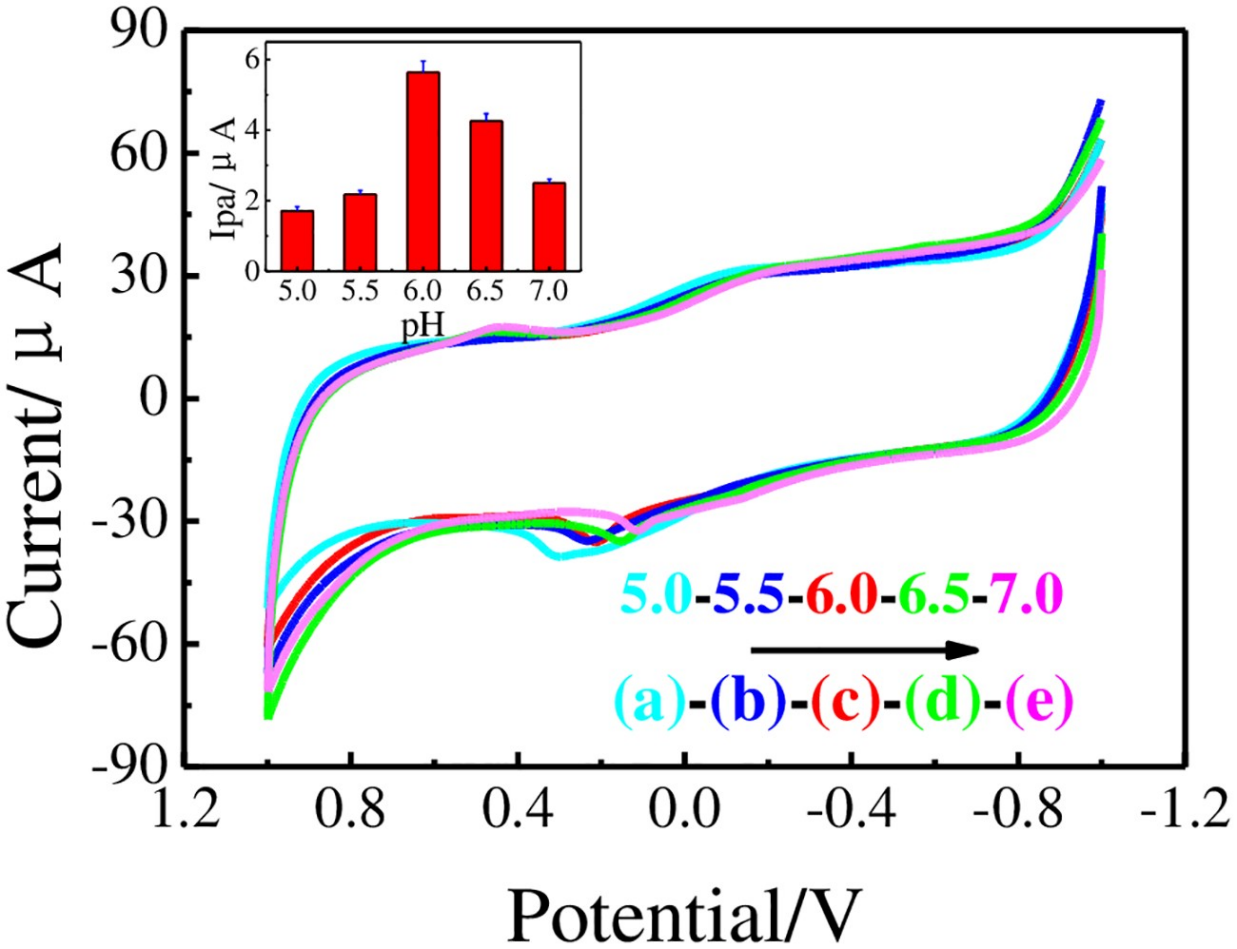
CVs of 20.0 μM DMY on GCE│rGO-Au/MIPs in PB varying pH levels 5.0 (a), 5.5 (b), 6.0 (c), 6.5 (d), and 7.0 (e). Insets: Ipa *vs*. pH.

Fig 8 shows CV responses of GCE│rGO-Au/MIPs electrode in different pH values PB containing 20.0 μM DMY. The Ipa increased gradually when pH values changed from 5.0 to 6.0 and decreased at the higher pH values of 6.5 and 7.0. The largest Ipa was obtained at pH value of 6.0. It can be interpreted that the adsorption capacity of DMY on imprinted membrane was strongest and the activity of DMY was highest. As the pH values increased, the peak potentials shifted negatively, which indicated the protons participated during the electrochemical reaction [29–31]. Therefore, pH = 6.0 PB was selected as supporting electrolyte.

### Detection of DMY

**Fig 9:**
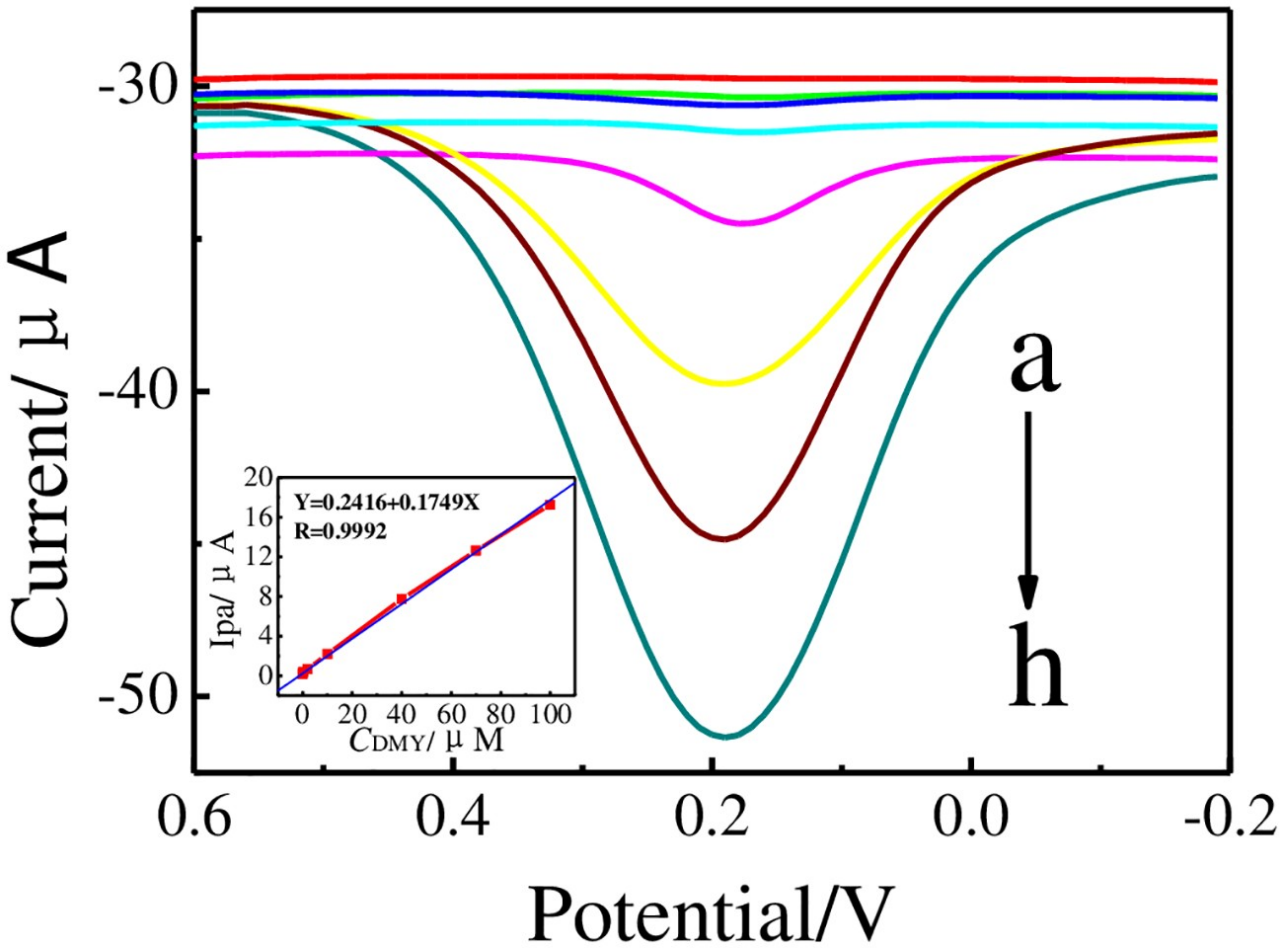
DPVs of GCE│rGO-Au/MIPs in PB containing 0 (a), 0.02 (b), 0.2 (c), 2.0 (d), 10.0 (e), 40.0 (f), 70.0 (g), and 100.0 (h) μM of DMY. Insets: the calibration curve of *C*_DMY_ *vs*. Ipa.

Under optimized experimental conditions, the DPV responses of GCE│rGO-Au/MIPs electrode recorded by different concentrations of DMY are demonstrated in Fig 9. The concentrations of DMY (*C*_DMY_) in a wide linear detection range (LDR) of 2.0×10^−8^ to 1.0×10^−4^ M are proportional to well-defined Ipa currents. The linearization equation is: Ipa(μA) = 0.2416+0.1749*C*_DMY_(μM) (*R* = 0.9992). A low limit of detection (LOD) obtained with the calculation is 1.2×10^−8^ M (*S/N* = 3).

Compared with some other DMY electrochemical sensors reported previously, the GCE│rGO-Au/MIPs electrode presented with the widest LDR and the lowest LOD (Table 1).

**Table 1:** Comparison of several typical DMY electrochemical sensors.

Electrode	Modifiers	LDR/M	LOD/M	Stability	Ref.
GCE	SWNTs^a^-nafion	1.0×10^−7^–1.0×10^−5^	9.0×10^−8^	7 days	[7]
GCE	DNA/En^b^/PGA^c^	4.0×10^−8^–2.0×10^−6^	2.0×10^−8^	7 days	[8]
GCE	Gr-nafion	8.0×10^−8^–2.0×10^−5^	2.0×10^−8^	56 days	[9]
GCE	rGO-Au/MIPs	2.0×10^−8^–1.0×10^−4^	1.2×10^−8^	56 days	This work

^a^ SWNTs: Single walled carbon nanotubes.

^b^ En: Ethylenediamine.

^c^ PGA: Polyglutamic.

### Reproducibility, stability, and selectivity

The reproducibility of GCE│rGO-Au/MIPs electrode was valued with 10.0 μM DMY. A relative standard deviation (*RSD*) of 3.25% can be calculated after five successive assays using the same modified electrode. Five independent modified electrodes were used to study the electrode-to-electrode reproducibility. The obtained *RSD* was 2.88%. The reproducibility of modified electrode is acceptable.

The stability of GCE│rGO-Au/MIPs electrode is defined as the DPV response towards 10.0 μM DMY remained higher than 90.0% of its initial current. The DPV response towards 10.0 μM DMY preserved at 90.3% of its initial current after 8 weeks storage in dry and ambient condition, demonstrating long-term stability of modified electrode.

**Fig 10:**
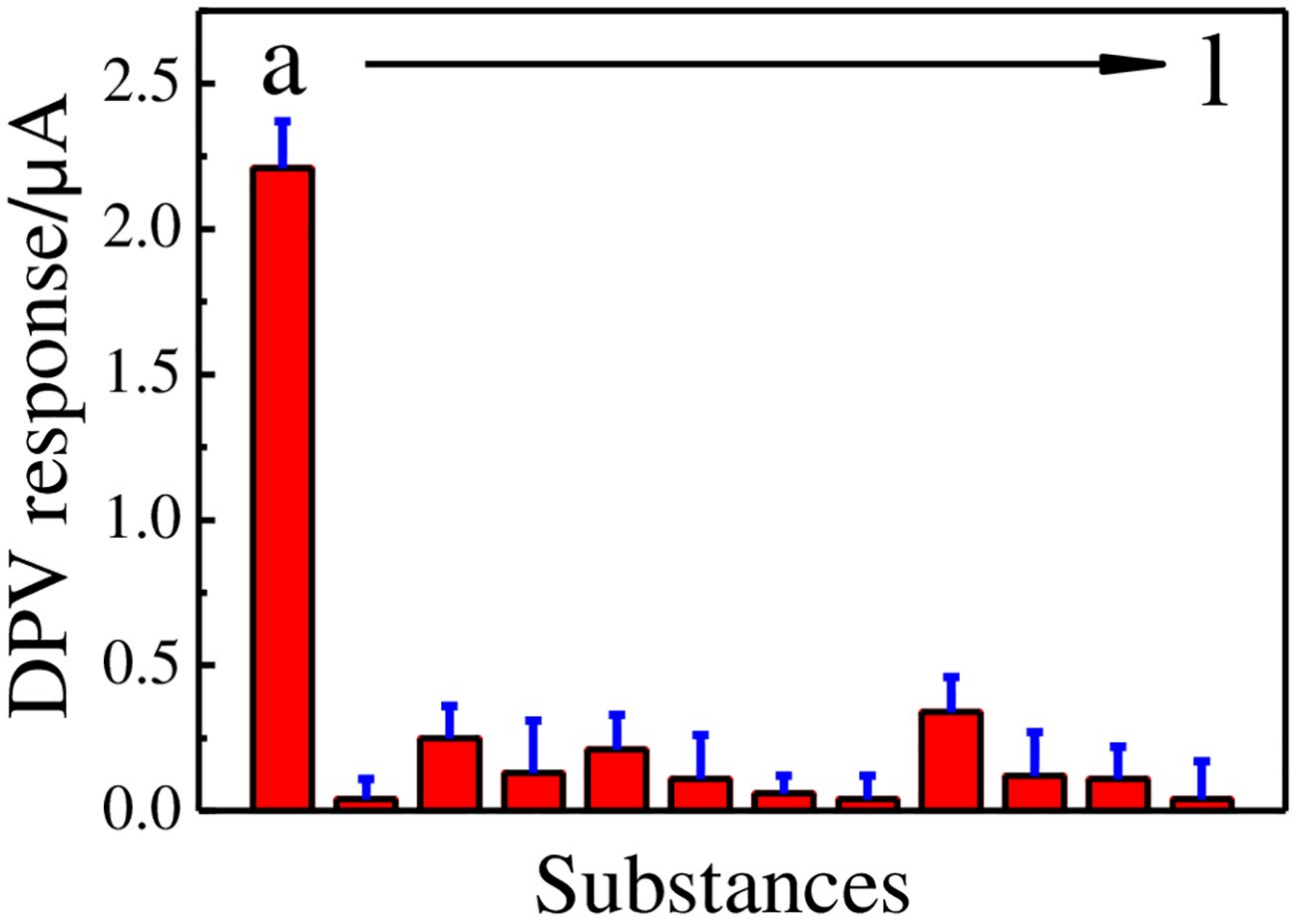
DPV responses of 10.0 μM of DMY (a), 0.1 mM of glucose (b), dopamine (c), uric acid (d), ascorbic acid (e), caffeine (f), glutamic acid (g), theophylline (h), rutin (i), quercetin (j), myricetin (k), and bisphenol A (l) on GCE│rGO-Au/MIPs.

The substrates including coexisting interferents, structurally analogues, and inorganic ions that may affect current responses of GCE│rGO-Au/MIPs electrode towards a fixed DMY concentration of 10.0 μM were investigated. The selectivity is defined as a relative error of response lower than 5%. No interferences were arisen by addition of 20-fold concentrations of Cl^-^, NO_3_^-^, K^+^, Na^+^, SO_4_^2-^, Fe^2+^, Zn^2+^, Mg^2+^, Ca^2+^, Mn^2+^, and PO_4_^3-^, 10-fold concentrations of glucose, dopamine, uric acid, ascorbic acid, caffeine, glutamic acid, theophylline, rutin, quercetin, myricetin, and bisphenol A (Fig 10). The results indicate good selectivity of modified electrode (Table 2).

**Table 2:** The selectivity coefficients (*k*) values of some analogues on GCE│rGO-Au/MIPs.

Substances	DPV responses/μA	*k*
DMY	2.21	-
Glucose	0.04	55.25
Dopamine	0.25	8.84
Uric acid	0.13	17.00
Ascorbic acid	0.21	10.52
Caffeine	0.11	20.09
Glutamic acid	0.06	36.83
Theophylline	0.04	55.25
Rutin	0.34	6.50
Quercetin	0.12	18.41
Myricetin	0.11	20.09
Bisphenol A	0.04	55.25

### Real samples detection

The pretreatments of Ampelopsis grossedentata samples and quantification of DMY by HPLC were followed the literatures [7, 8]. Briefly, a certain amount of sample was mixed with water (liquid-solid ratio = 10:1), boiled for 60 min and filtered. The filtrate was collected and stored in refrigerator. The precipitates were dispersed in ethanol and analyzed as real samples. The quantification of DMY by HPLC was performed on a 4.6×150 mm C18 column combined with an ultraviolet-vis detector (290 nm). The mobile phase with a flow rate at 1.0 mL**·**min^-1^ is 0.1% phosphate/methanol solution (*v*/*v* = 70:30). The contents of DMY in real samples by calculating from the linear regression equation with standard solution are displayed in Table 3. Furthermore, the results obtained by HPLC and standard addition methods displayed no significant statistically difference, confirming the reliability of proposed method.

**Table 3:** Determination results of DMY in Ampelopsis grossedentata by DPV and HPLC.

DPV (n = 3)						HPLC (n = 3)	
Sample	Amount found (mg·L^-1^)	*RSD* (%)	Added (mg·L^-1^)	Total found (mg·L^-1^)	Recovery (%)	Amount found (mg·L^-1^)	*RSD* (%)
1^#^	2.78	2.53	2.00	4.70	96.0	2.75	3.12
2^#^	2.73	4.02	2.00	4.66	96.5	2.75	3.52
3^#^	2.64	3.44	2.00	4.72	104.0	2.74	2.65

## Conclusions

In summary, the sensitive, selective, and reliable electrochemical sensing platform based on double-layered membranes modified GCE for DMY determination was fabricated. The electrochemical reduction and polymerization strategies involved in the electrode modifying procedures combined the advantages of in-situ, controllable, facile, rapid, high usage, and environmental friendly. The fabricated modified electrode also showed superiorities of long-term stability, wide LDR, and low LOD compared with several other DMY electrochemical sensors due to the synergistic effects originated from composites. The practical applications of electrochemical sensor were confirmed by measuring real samples. The methodologies in preparation of MIPs-based composites and fabrication of sensing platform are universal, which possess widespread applications in the field of Chinese traditional herbal medicines analysis and quality monitoring.
